# 

**Published:** 1889-10-05

**Authors:** 


PRICE ONE PENNY.

No. 158, Vol. VII.—October 5th, 1889.

Registered at the General Post Office as a Newspaper.]

Per Annum, 6s. 6d., post free.

Monthly Parts, 6d.; post free 7½d.

Ditto per Annum, 7s. 6d., post free.

